# The Demographic Diversity of Food Intake and Prevalence of Kidney Stone Diseases in the Indian Continent

**DOI:** 10.3390/foods8010037

**Published:** 2019-01-21

**Authors:** Manalee Guha, Hritwick Banerjee, Pubali Mitra, Madhusudan Das

**Affiliations:** 1 Department of Zoology, University of Calcutta, 35 Ballygunge Circular Road, Kolkata 700019, India; manalee.guha@gmail.com (M.G.); hritwick.electrical@gmail.com (P.M.); 2 Department of Biomedical Engineering, Faculty of Engineering, 4 Engineering Drive 3, National University of Singapore, Singapore 117583, Singapore; biehb@nus.edu.sg

**Keywords:** food intake, food diversity, kidney stone disease, social epidemiology

## Abstract

Food intake plays a pivotal role in human growth, constituting 45% of the global economy and wellbeing in general. The consumption of a balanced diet is essential for overall good health, and a lack of equilibrium can lead to malnutrition, prenatal death, obesity, osteoporosis and bone fractures, coronary heart diseases (CHD), idiopathic hypercalciuria, diabetes, and many other conditions. CHD, osteoporosis, malnutrition, and obesity are extensively discussed in the literature, although there are fragmented findings in the realm of kidney stone diseases (KSD) and their correlation with food intake. KSD associated with hematuria and renal failure poses an increasing threat to healthcare infrastructures and the global economy, and its emergence in the Indian population is being linked to multi-factorial urological disorder resulting from several factors. In this realm, epidemiological, biochemical, and macroeconomic situations have been the focus of research, even though food intake is also of paramount importance. Hence, in this article, we review the corollary associations with the consumption of diverse foods and the role that these play in KSD in an Indian context.

## 1. Introduction

Kidney stone diseases (KSD) and associated research have become widespread, with the correlation between genetic predisposition and calcium-based kidney stones being an early observation. Furthermore, in the USA, there was a sharp rise in KSD, i.e., nearly 200%, in the years between 1964 to 1972 [1]. In the same way, European countries, except Scotland (3.83% in 1977 to 3.5% in 1987), Germany, Spain, and Italy, have also shown an increasing trend in KSD prevalence in recent decades [2,3,4,5]. Along with these geographical boundaries, Japan and some parts of Iran, USA and other countries soon started investing money into research on KSD, focusing on general patterns relating to the age population of both men and women [6,7,8]. In conducting these studies, it was found that KSD is prevalent, with nearly 35% of the control group affected with hypercalcaemic nephrolithiasis disorder [9]. Along with this, reports have holistically demonstrated that monozygotic twins (32.4%) have approximately 15% higher rates in comparison to dizygotic twins (17.3%; *P* < 0.001) [10]. To further elaborate on this paradigm, reports have aptly depicted, in a Canadian context, that even though the presence of dent disease and hypophosphatemic rickets with hypercalciuria has been observed, there is still a strong relationship with ancestry and genetic patterns [11]. Furthermore, to conclude this hypothesis, they have to counteract several genetic analysis approaches, namely, encoding vitamin D receptor (VDR), calcium-sensing receptor (CaSR), 25(OH)D 1α-hydroxylase, osteocalcin, uromodulin, and osteopontin among others [12]. On the other hand, studies portraying gender and age as the principle parameters have revealed that Iran and USA are the peak KSD locations for people aged 40–49 years, while the greatest prevalence among people aged 50–59 years is shown in Japanese women [1]. The data illustrated a similar pattern in Japan and USA for the male group in the 40–49 age bracket, and Iran followed, with a different trend [13]. This initial investigation leads us to conclude that it is increasingly unscientific to extrapolate KSD patterns based on age and sex in different geographic locations. Therefore, the researchers were challenged to create a set of new parameters to fine-tune more realistic solutions in the realm of KSD and its prevalence.

Diet is an integral part of renal accumulation and thus filtration, which in turn affects the absorption and bodily homeostasis of renal stone occurrence [14,15]. The epidemiology differs in accordance with different geographical regions and social constructs. Within this context, food habits have been proposed as one of the major risk factors in renal stone formation, as a form of epidemiology for urine composition [16,17]. Food patterns are among the major factors for renal stone formation, and stone material deposition can be managed by regulating food intake. In the context of the Indian diet, there is a collection of many tastes and flavors, from the color-rich food of Rajasthan, to the spicy food of Punjab, and from the slightly sweet, oil-based food of Gujarat, to the southern, slightly sour seafood. All of these foods are found in this land of paradise. Several communities in the country are vegetarian, although there is still a large range of rich non-vegetarian recipes. Increases in urinary calcium excretion are strongly related to the consumption of animal proteins, with a consequent reduction in urinary pH and citrate excretion, which are the basis of stone formation. Due to this food pattern in Indian culture, we believe that it is of paramount interest to describe the nutritional factors causing renal stone formation and the immediate effects thereof.

## 2. Diverse Food Habits in India

The era of rapid globalization and packaged market products has shifted the equilibrium of nutrition in India. Urban areas in India have embraced more packaged foods, which have led to increased body mass and premature obesity. In this realm, Indian cooking reflects thousands of years of history, leading to the diversity of flavors and the innumerable regional dishes found in the country [18]. Diversified food habits range in different parts of India, with different geographical areas spanning from the Rajasthan desert to the Madhya Pradesh forest, or from the Maharashtra seashore to the Jammu and Kashmir Mountains [18]. For the ease of description, Indian cooking is classified principally into the North Indian, East Indian, West Indian, and South Indian cuisine, based on the similarity and differences in the nature of these cuisines. North Indian cuisines are distinguished by their heavy use of dairy products and the prevalence of flatbreads, such as roti and paratha, baked in clay ovens, over rice dishes. Even though parts of Western India, such as Gujarat and Rajasthan, are predominantly vegetarian, the remaining cuisines have their fair share of meat or animal proteins, owing to their associations with the Muslim incursion into India. South India can boast a bewildering range of regional cuisines. Therefore, with cuisines ranging from the rich northern style Mughlai cooking of the pre-dominantly Muslim dominated Hyderabad to the simple vegetarian dishes of Tamil Nadu, from the seafood, kebabs, and puris of Maharashtra to the strong Portuguese-influenced cuisine of Goa, from the coconut-based recipes of Kerala and the Malabar fish dishes to the unique cuisine of a shrinking Franco-Indian population in Pondicherry, South Indian food habits encompass a wide spectrum of culinary options [18]. Socio-demographic, macroeconomic, and lifestyle factors in the coastal part of Karnataka and Kerala illustrate the close proximity between observed dietary habits and food styles. The transition from South Indian to Eastern Indian food habits encompasses cuisines from the states of West Bengal and Orissa to the Northeastern states. The staple foods of this region are largely based on rice and wheat. East Indian food habits have a good balance of vegetarian and non-vegetarian dishes, with fish curries being the cornerstone of non-vegetarian dishes. Steaming and frying are popular methods of cooking. Therefore, to quantify the diversity of Indian food styles and their relationship with KSD, this review article investigates possible methods for curtailing the prevalence of KSD, which is associated, directly or indirectly, with food consumption.

### 2.1. Stone Forming Area in India

**Figure 1:**
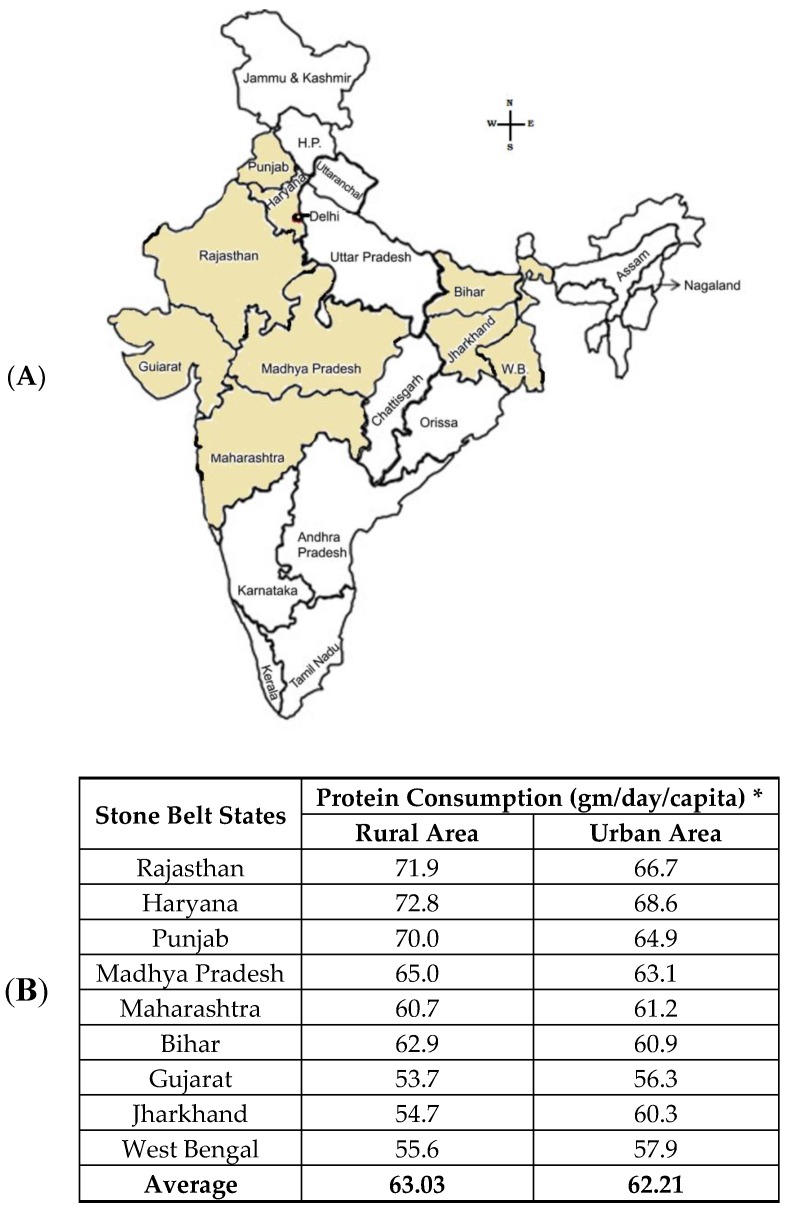

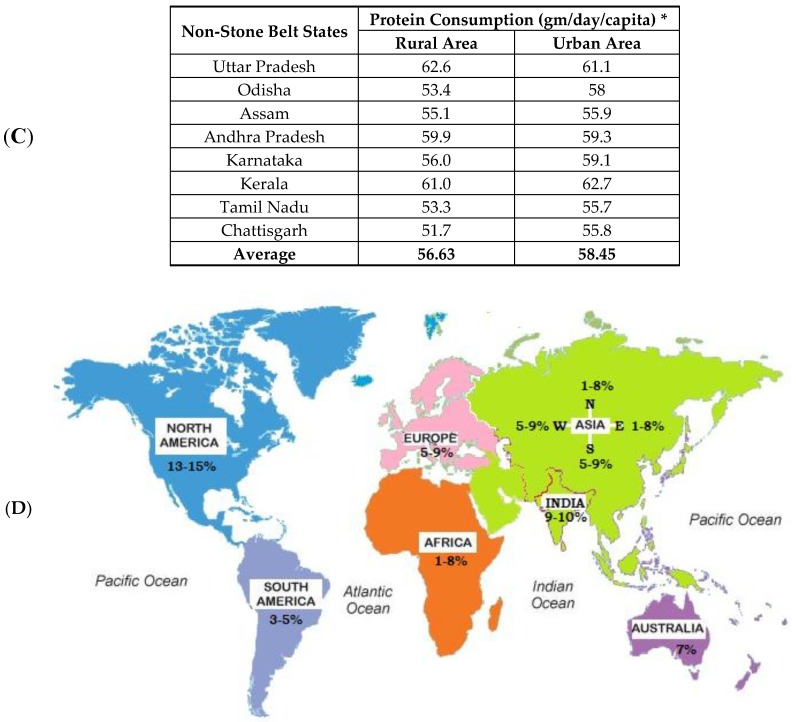
Stone belt Area: (**A**) Major kidney stone prevalent states in the Indian continent. (**B**) Animal protein consumption per gram per day per capita in stone belt Indian states, which leads to KSD (* Ministry of Statistics and Programme Implementation 2012). (**C**) Non-stone belt Indian states are also depicted with the rural and urban population and the animal protein consumption per gram per day per capita. (**D**) Prevalence rates of kidney stones in a global platform, for comparison.

In the context of India, KSD is prevalent, with an expectancy of 12% in a total population reported to be prone to urinary stones [19]. Of this 12%, 50% of the population are severely affected by renal damage, which even leads to a loss of kidneys [19]. Unlike in South India, where a few reported percentages affected by Urolithiasis, in North India, there is a steep 15% of the population within the realm of KSD [20]. Thus, considering the prospects of the kidney stone belt, which are affected by KSD in India, a proper corollary needs to be established [20]. This stone belt occupies areas of Maharashtra, Gujarat, Rajasthan, Punjab, Haryana, Delhi, Madhya Pradesh, Bihar, and West Bengal (Figure 1). In these regions, the frequency of the prevalence and recurrence rate of renal stone is high in most of the members of a family.

### 2.2. Food Habits with Stone Formation

The Indian food habit has been considered a widely recognized risk factor of kidney stone formation [21]. An increase in calcium excretion after a load of protein was stated by many studies [22,23]. The increased consumption of animal products leads to higher calcium, oxalate, and phosphorous in the urinary tract [24,25,26]. These are the reasons for stone formation, initially in the form of insoluble calcium oxalate or calcium phosphate crystals. Proteins also increase uric acid generation, which may end up in stone formation [25,26]. High carbohydrate and lipid consumption has been shown to have similar changes [27]. The presence of a high amount of salt in fast food, especially in industrialized countries, causes higher calcium in the kidneys [28]. Conversely, a low calcium diet is considered to be a risk factor, as it increases the intestinal absorption of oxalate [29]. Citric acid, potassium, and magnesium act as negative regulators of stone synthesis [25]. For this reason, the inadequate intake of fruits and vegetables are considered as risk factors for stone synthesis, although some oxalate-rich fruits, such as berries, chikoos, and vegetables, such as tomatoes, spinach, and beets, are still of some risk [30,31]. Vitamin C, when administered in higher quantities into the human body, is reported to cause kidney stones in some cases [32]. It is inferred that Vitamin C gets converted to oxalates [33]. However, some studies reported that oxalate excretion is not closely related to dietary intake [34]. There are a number of comparative studies of stone formers and the healthy control of dietary habits [27,29], but the results are contradictory. Most of these studies support the relation between food habits and kidney stones, although the opposite result has also been found [35]. Thus, the development and progress of the disease is not clear. In this connection, Table 1 corresponds to reports conducted in the different geographical study populations to strengthen the correlation of food intake and its impact on KSD.

#### 2.2.1. Protein

A high intake of protein, especially animal protein, is responsible for the relatively high prevalence of stones [23,49,53]. Animal protein containing purines are precursors of uric acid stones [54]. Amino acids, such as glycine, tyrosine, and tryptophan, convert into oxalate, which is a very common component of kidney stones [55]. It also causes renal acid excretion, calcium reabsorption, increased urinary calcium excretion, and an increased renal reabsorption of citrate, which ultimately leads to kidney stone formation [49]. This protein makes a good contribution to forming a bridge between calcium (increased calcium) and uric acid (decreased citrate) stones by its activity. While a balanced amount of protein intake is required to ignite metabolism, greater consumption, on the other hand, increases the burden in the kidney and liver [56]. To support the fact of protein consumption and associated kidney stones, reports have shown that meat consumption in the developed nation is three times higher than that in developing ones, such as Asia between 1970 and 1990 [57].

#### 2.2.2. Calcium-Rich Food

Nishiura (2002) demonstrated a comparative study of the control individuals and stone former individuals in relation to the oral consumption of a calcium diet, with urinary excretion. In stone formers, there is a dependency of urinary calcium excretion on diet, whereas in controls, there is a variation in calcium excretion corresponding to diet [34]. On the contrary, it has been shown that lower calcium intake provides a higher risk of stone formation than higher calcium intake [58]. Calcium-rich cereals, such as ragi, rajma, soybeans, or dairy products, are the main ingredients of a regular diet in India, especially in the stone belt region [18]. Conversely, the intake of calcium supplements outside meals causes an increased risk of stone formation in patients taking calcium supplements [34]. Calcium intake outside meals results in a different effect than does calcium intake with other nutrients [45].

#### 2.2.3. Carbohydrate-Rich Food

Calcium stone formers exhibit an enhanced urinary calcium excretion of dietary content containing high carbohydrates in comparison to healthy controls [59]. Carbohydrates reduce the reabsorption of calcium at the level of the distal tubule, but subsequent studies have shown that glucose in a high concentration can enhance the intestinal absorption of calcium [60]. In addition, fructose increases the urinary excretion of calcium and oxalate, both of which are important risk factors for calcium stones. Low urinary pH, which is a major reason behind uric acid formation, is the trailing step of insulin resistance due to excessive fructose intake [61]. It was reported that uric acid synthesis is upregulated due to a single carbohydrate component, fructose [62]. In India, the diet of northern and eastern regions contains the maximum sucrose content of everyday life [63].

#### 2.2.4. Sodium and Potassium

The modification of sodium by cutting down the daily intake of salt is advised for reducing kidney stone recurrence [64]. Changes in the composition of urine, i.e., increased calcium or decreased citrate, is attributed to increased sodium in the diet [14]. It is shown that sodium can greatly affect the urinary excretion of calcium, i.e., 25 mmol/day increases in urinary sodium cause an increase of 0.6 mmol/ day in urinary calcium [65]. Sodium and calcium excretion in the urine are well correlated, and this is shown in some studies [52,66]. Potassium also regulates the value of urinary calcium in the body [67]. In one study, it was reported that in healthy subjects’ diet, with normal sodium quantity, dietary potassium deprivation is associated with an increase in urinary calcium excretion [68]. The regular salty food pattern is one of the reasons behind kidney stone formation in India [69,70]. In contrast, sodium and potassium also increase urinary volume and pH, which is required in cystine lithiasis [71].

#### 2.2.5. Oxalate-Rich Food

The dietary oxalate and the metabolism of vitamin C both cause oxalate [38,39,40]. Urinary oxalate excretion derives from metabolism, but 10–50% comes from dietary oxalate [38]. In Western countries, the intake of oxalate ranges between 100–300 mg/day, and approximately 5–10% of the total is absorbed in the intestine [72]. Intestinal absorption depends on the form in which it is consumed, i.e., soluble or insoluble, and on its interaction with other food materials [73]. The main sources of dietary oxalate are relatively few: spinach (45%), potatoes (10%), cold cereal (4%), nuts, coffee, and tea account for about 70% of all dietary oxalate [30,31]. Some studies claim that renal stone formers consume more oxalate than healthy controls, and there are some studies where the oxalate quantity of the diets is not the only reason for renal stone formation [34]. In Table 2, we compiled diverse food intake in different Indian regions, i.e., central (Uttar Pradesh, Uttarakhand, Madhya Pradesh, Chhattisgarh), eastern (West Bengal, Tripura, Sikkim, Odisha, Nagaland, Mizoram, Meghalaya, Manipur, Jharkhand, Bihar, Assam, Arunachal Pradesh), northern (Punjab, Jammu and Kashmir, Himachal Pradesh, Haryana), western (Rajasthan, Maharashtra, Gujarat, Goa) and southern (Telangana, Tamil Nadu, Kerala, Karnataka, Andhra Pradesh) regions, and the macromolecular content related to KSD (Table 3).

## 3. Mechanism of Different Types of Stones According to Food Habits

### 3.1. Impact of Food on the Mechanism of Stone Formation

Kidney stones are named according to the names of the crystals that make up the hard part of the stones: calcium oxalate, calcium phosphate, uric acid, cysteine, and struvite. In India, calcium oxalate and calcium phosphate stones are predominant, whereas reports of uric acid and cystine stone are very few [74]. Struvite stones are not under consideration, as they are formed by bacteria in urinary tract infections and are generally not found in the Indian population [21].

#### 3.1.1. Calcium Stone

**Figure 2:**
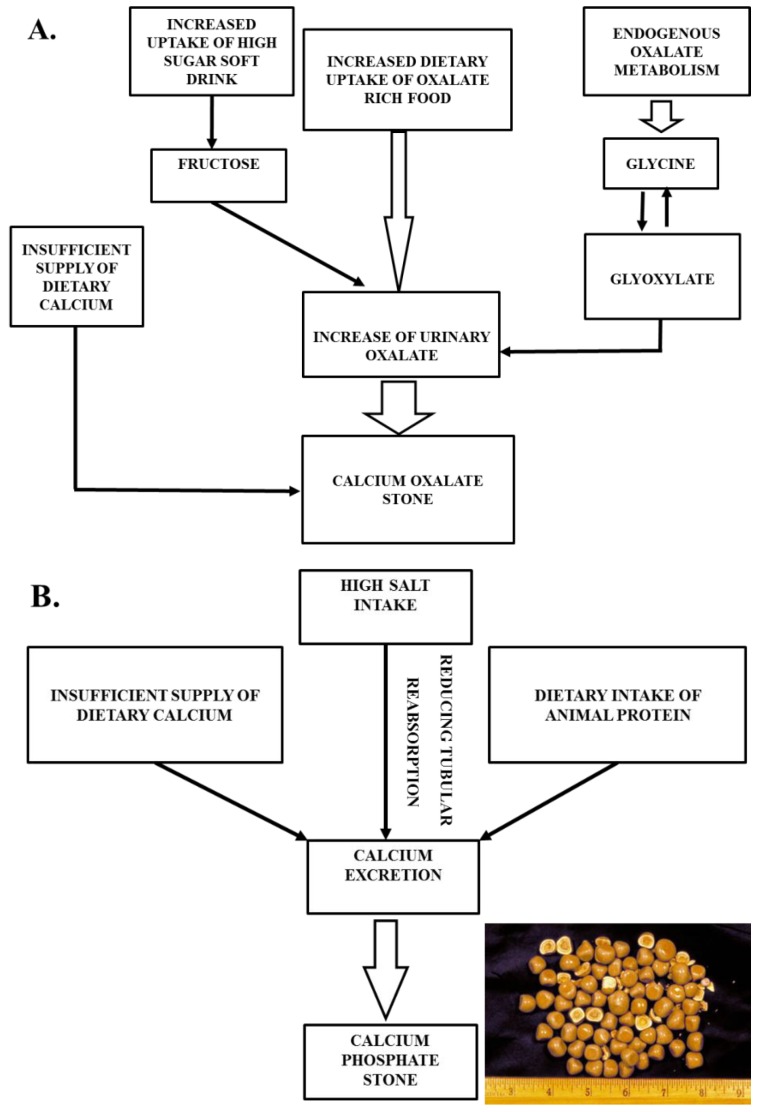
Calcium stone formation with food habits: (**A**) Calcium oxalate stone formation, and (**B**) calcium phosphate stone aggregation.

Calcium is the major element of about 80–90% of all urinary stones [75]. They are usually made of calcium oxalate or calcium phosphate or mixtures of them detected in chemical or infrared spectrometric analysis [76]. Calcium phosphate may solidify in the renal interstitium and, later, on a papillary surface along with calcium oxalate [77]. Many studies reported a derivation of 10–50% of the urinary oxalate in diets rich in dark-green leafy vegetables, spinach, beets, beans, cereals, dietary ascorbic acid, glycine-rich food, such as animal proteins, chocolate, black tea, etc. [2,38,39,40,73,78]. The protein breakdown product, glycine, oxidizes to glyoxylate in a metabolic pathway, which is the precursor of oxalate, a major stone component [79]. It has been shown that the overconsumption of animal protein creates an observable increased rate of urinary calcium (23%) and oxalate (24%) [80]. High fructose consumption from soft drinks is associated with an increased risk of hypercalciuria, hyperoxaluria, and hyperuricosuria [39,81]. In an experiment, higher urinary calcium excretion occurred in rats fed high-fructose diets, unlike in rats fed high-starch diets [82,83]. An insufficient supply of dietary calcium is also a notable risk factor for both calcium oxalate and phosphate stone formation [34,45]. High salt intake has been associated with elevated urinary calcium excretion, because it reduces tubular reabsorption, which is an output of the free particle model on crystallization [84]. These high concentrations of calcium in the urine combines with oxalate and phosphorus to form stones (Figure 2).

KSD is mostly accompanied with hypercalciuria in 30–60% patients due to high intestinal calcium absorption [85]. In hypercalciuria, calcium stimulates the supersaturation of mineral crystallization and makes the obstacles of stone inhibitory factors (citrate, glycosaminoglycans) by binding with them [86]. Other events, such as bone resorption and renal leakage, play a positive role in implicating hypercalciuria manifestation [87]. By reducing calcium intake, the heights of calcium excretion are manageable at a certain level [88]. Thus, dietary management should be required for the regulation of hypercalciuria. A diet-based study demonstrated that a lower intake of animal proteins and salts, with optimum calcium intake, have a great impact on reducing the chance of stone recurrence [52,66]. Overtaking salts and proteins increases urinary calcium excretion by nearly 23%, and the outcome is kidney stone formation [52]. From a rat model experiment with elevated urinary calcium excretion, fructose was also found to have a significant role [89].

#### 3.1.2. Uric Acid

**Figure 3:**
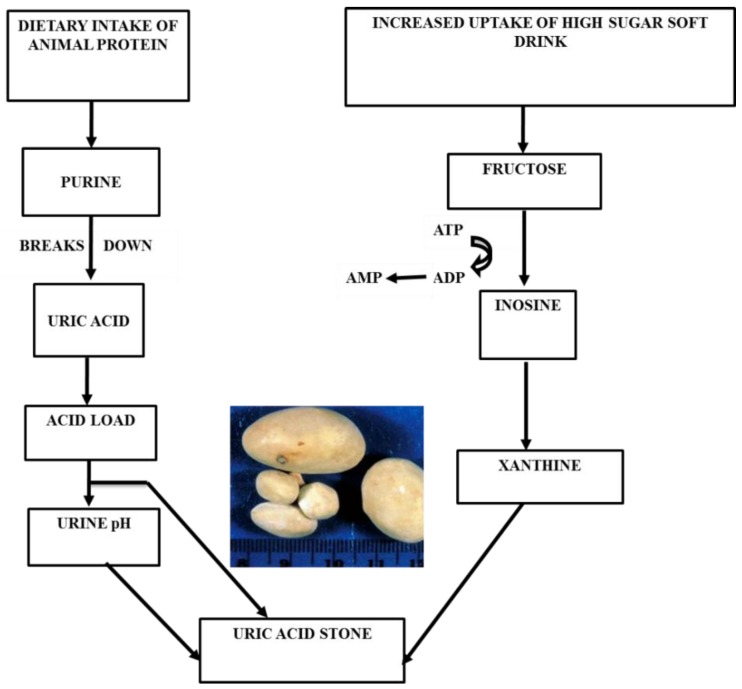
Uric acid stone formation with food habits.

Uric acid stones constitute nearly 5–10% of urinary stones [90]. Low urine pH is often associated with this type of stone [22]. Urinary uric acid solubility decreases by approximately 185 mg/dL when urine pH drops from 7 to 5. At a higher pH, 95% of uric acid is in its soluble urate form, and at a lower pH solubility, it decreases in most of the uric acid [91]. Excess uric acid is excreted through urine, and hyperuricosuria is caused by a purine-rich diet, a precursor of uric acid (Figure 3) [90]. Foods containing high protein, especially animal protein, such as poultry things, eggs, meat, sea fish, and some plant products, such as seeds and nuts, are the highest sources of purines [92]. It was reported that a daily increase of animal protein significantly increased uric acid excretion by 48% [93]. Another factor, sweet drinks with a high fructose level, is related to an increased risk of renal stones. In one study, it was shown that the overconsumption of fructose results in a rapid rise in serum uric acid through increased purine synthesis [89]. In the metabolism pathway, fructose breaks into insonine and xanthine with the help of energy driver ATP. This xanthine ultimately promotes uric acid formation using ADP as a substrate [94].

#### 3.1.3. Cystine Stone

**Figure 4:**
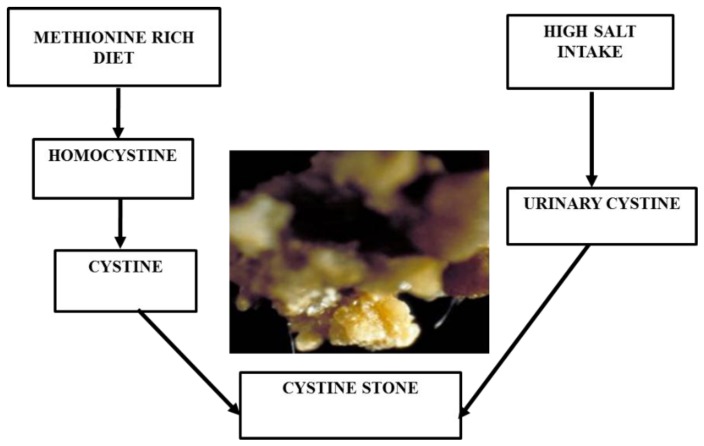
Cystine stone formation with food habits.

Cystine stones are very rare, constituting only 1–2% of urinary calculi [95]. These are formed in people who have a tendency of excessive cystine leakage from the kidneys into the urine [96]. Maintaining cystine concentration in urine below 200 to 300 mg/L is the best medical method for avoiding this type of stone [97]. A high liquid substance is required for producing at least 3 L of urine a day to decrease the high level of urinary cystine [98]. In addition, potassium citrate is usually taken as a drug for reducing renal acidosis [99]. In food, animal proteins containing methionine, such as meat, break down into cystines, which increases the urinary cystine level in the body [100]. It has been shown that urinary cystine excretion can be reduced to 34% by consuming a very low protein diet (nearly 20 g/day) [101]. Overtaking sodium has a high impact on the manifestation of this disease, since it promotes the excretion of cystines (Figure 4) [102].

## 4. Food Diversity and Nutritional Effects in Indian Population

Over-nourishment and undernourishment are both great burdens for society. On the one hand, the excess intake of proteins, carbohydrates, and oxalate-rich foods enhances the occurrence of renal stones [23,59,61,62,72]. On the other hand, increased protein breakdown and protein undernutrition are familiar in chronic kidney disease (CKD) patients [103]. Thus, it is evident that the presence or absence of protein has an observable influence on these two kidney diseases. Several studies have suggested that CKD is a recognized issue among stone formers [104,105,106]. Renal stone is a risk factor of an impaired kidney function, and an important clinical parameter, the serum creatinine level, should be monitored in the follow-up [107]. In a population study from the US, it was indicated that elevated serum creatinine levels cause a nearly 25–44% increased risk of CKD in stone formers [108]. Recurrent stone formers might accumulate incremental kidney injuries with each stone event. Therefore, treatments to prevent kidney stone recurrence may be beneficial for delaying CKD progression, especially because kidney stone events are associated with reductions in glomerular filtration rates and increases in proteinuria [105,106,107]. During a mean of 8.6 years of follow-up, stone formers had an increased risk of being clinically diagnosed with CKD [108]. A protein-rich diet may increase serum creatinine levels, which has an impact on changes in the glomerular filtration rate [109]. Interestingly, there are multiple reports on protein energy malnutrition, which occurs during the CKD, especially in the mature stages (3–5), and the risks of mortality are high due to the occurrence of protein malnutrition at the time of dialysis [110]. Many studies have also shown that CKD patients have a lot of resting energy expenditure in comparison to normal individuals, and during dialysis, this expenditure is further increased [111,112]. For this reason, they require more energy, especially protein. In conclusion, food management is a very important tool for maintaining the health of the kidney.

**Figure 5:**
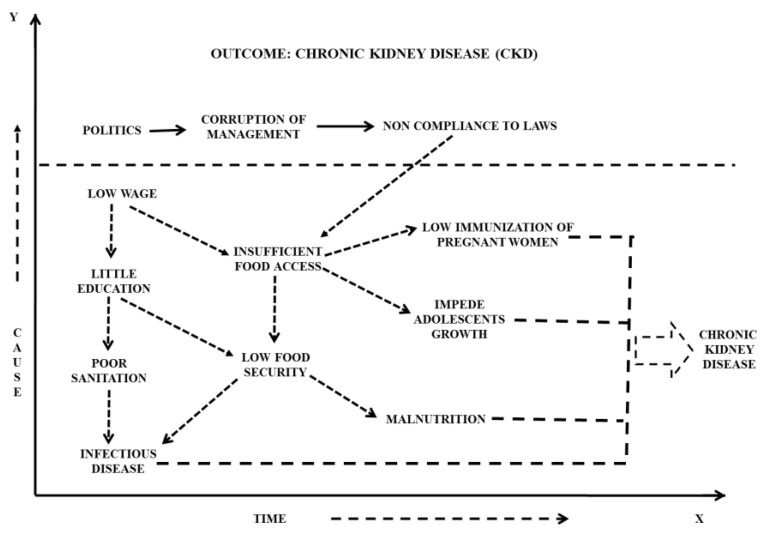
Diagrammatic representation of a causal conceptual model in the Indian scenario. An example where societal factors, such as economy, political view, and education, affect the health and wellbeing of the poorer class of the Indian population.

In the Indian scenario, where mostly there are people under the poverty line, income plays a major role in determining the status of life and food intake in general [101]. This poor socioeconomic position is associated with chronic malnutrition, since it inhibits the purchase of essential nutritious foods for growth and development, as the price rates are not increasing with income proportionately [113]. Thus, there are many indirect pathways, such as poor healthcare, malnutrition, abstemious food intake, that lead to a CKD and many other medical conditions (Figure 5) [114]. Prenatal cases, where calcium intake and nutritional levels need to be properly maintained, have frequently been the underprivileged. In this section, we have given an introductory causality model, revealing causes that very much exist in India, in the realm of nutrition, food intake, and their impact on human lives.

## 5. Concluding Remarks

KSD is a rising concern, a major healthcare burden, and is associated with hematuria and renal failure. The risk of renal stone varies from 1–5% in Asia, 5–9% in Europe, 10–15% in the USA, and 20–25% in the Middle East [115]. Dietary therapy can be one of the promising solutions for minimizing the cases of recurring kidney stone formation and hence a better quality of life. Protein and carbohydrates are the most dominant food contents in India. Rural as well as urban areas of the stone belt region consume a high amount of protein in comparison to other regions. These food habits are one of the main reasons behind the prevalence of KSD in India. Thus, awareness of the health concern and optimized food therapy can potentially curtail the cost of hospitalization and enhance compliance in general. Vis-à-vis dietary control and the insufficient understanding of the molecular and genetic basis of pathogenic mechanisms remains a critical barrier to early detection and treatment. Stone formation is mostly attributed to two mechanisms: (1) renal calcium leak, excessive absorption, bone resorption/formation imbalance; and (2) mineralization. Dietary factors have been widely recognized as one of the primary risk factors of kidney stone formation [59]. On the other hand, the parathyroid hormone primarily modulates calcium balance. It increases calcium excretion in the kidney. VDR regulates calcium homeostasis by affecting bone resorption and calcium absorption. CLDN-14, a tight junction protein, decreases Ca^2+ ^permeability, whereas matrix gla protein (MGP) regulates calcification [76]. Secreted Phosphoprotein 1 (SPP1) prevents renal stone formation by decreasing the aggregation of crystals and binding to the renal epithelial cells [115]. These genes are significantly associated with KSD in the Indian population [76]. These are the reasons for the initial stone formation in the form of insoluble calcium oxalate or calcium phosphate crystals. Most of these genetically inspired reports support the relation between food habits and kidney stones, although stratified contradictory reports also exist. Thus, conclusively, there is a high demand for a better understanding of the correlation between food intake and CKD, and hence quantified research and associated case studies need to be established in the near future.

## Figures and Tables

**Table 1:** Impact of food content and the prevalence of kidney stone diseases (KSD) in some different zones.

Food Content	Impact on Stone Formation	Studied Zone	Reference
Dietary oxalate	Intestinal hyperabsorption of oxalate increased urinary oxalate excretion	Western part of India	Pendse et al., 1986 [36]
		Germany	Hesse et al., 1993 [2]; Siener et al., 2003 [37]
		North Carolina, USA	Holmes et al., 2001 [38]
		Italy	Meschi et al., 2004 [31]
		Boston	Taylor and Curhan, 2007 [39]
		Eastern India	Mikawlrawng et al., 2014 [21]
Dietary ascorbic acid	Increases urinary oxalate excretion	New York	Urivetzky et al., 1992 [40]
		Italy	Trinchieri et al., 1998 [41]
		Washington	Massey et al., 2005 [42]
		Sweden	Thomas et al., 2013 [32]
		Boston	Ferraro et al., 2016 [43]
High dietary calcium	Reduces calcium oxalate stone formation	France	Bataille et al., 1983 [44]
		Boston	Curhan et al., 1993 [35]
		Germany	Siener et al., 2003 [37]
High intake of carbonated beverage	Increases urinary oxalate	Boston	Curhan et al., 1997 [45]
		Women of Omaha	Heaneyand Rafferty, 2001 [46]
		Netherland	Asselman and Verkoelen, 2008 [47]
		Boston	Taylor et al., 2009 [15]
		North Carolina	Saldana et al., 2007 [48]
Protein rich diet	Increases acid load in the kidney increases risk of stone formation	Boston	Curhan et al., 1997 [45]
		Chicago, USA	Reddy et al., 2002 [49]
	Reduce the bone’s ability to absorb calcium	Switzerland	Nguyen et al., 2001 [50]
	Increases urinary calcium	Italy	Borghi et al., 2002 [14]
High intake of sodium	Increases urinary calcium	Northern India	Awasthi and Malhotra, 2013 [51]
		Post-menopausal women of Korea	Park et al., 2014 [52]
		Southern India	Sofia et al., 2016 [19]

**Table 2:** Different geographic regions and food habits of India.

Indian Part	Food *	Protein %	Calcium %	Carbohydrates %	Sodium-Potassium %	Oxalate %	Dominant Food Content Related to KSD
Central	Mughlai	10–18	7	20–56	1	-	Protein
	Mushroom	6	1	-	9	-	
	Bamboo shoots	5	1	1	15	-	
	Pickle	-	-	-	50	-	
East	Fish	44	1	-	2–10	-	Protein and Carbohydrate
	Meat	52	0	-	2–12	-	
	Egg	26	5	-	3–5	-	
	Rice	5	1	9	1	-	
	Potato	4	1	10–20	6–12	1	
	Tomato	1	1	1	-	1	
	Spinach	5	9	1	3–15	1	
	Chives	6	9	1	2–8	-	
	Dairy	3	8	1	2–4	-	
North	Kidney bean	48	14	20	20–40	-	Protein and Carbohydrate
	Wheat	28	3	23	12	-	
	Corn	18	-	24	1–8	-	
	Mughlai	10–18	7	20–56	1	-	
	Paratha-Saag	10	11	30	1	-	
	Tomato	1	1	1	2–5	1	
	Legume	10	2	4	0–6	1	
	Dairy	3	8	1	2–4	-	
West	Seafish	30–40	-	-	2–10	-	Protein
	Crabs	36	9	-	7–15	-	
	Nut	40	11	7	11	1	
	Rice	5	1	20–28	-	-	
	Coconut	6	13	-	1	-	
	Sweets	3	8	1	2–4	-	
South	Dosa/Idli	3	6	23	-	-	Protein and Carbohydrate
	Grains	26	10	14	6–15	-	
	Fish	44	1	-	2–10	-	
	Meat	52	0	-	2–12	-	
	Coconut	6	13	-	1	-	
	Pickle	-	-	-	50	-	

* Some of the most common foods consumed in different parts of India.

**Table 3:** Various macromolecules/nutrients and their effect on the level of KSD.

Macromolecules/Nutrients	Potential Level in KSD
Protein rich food	High
Calcium rich food	High, sometimes low
Carbohydrate rich food	High
Sodium Potassium	High
Oxalate rich food	High
